# A Case of Acute Pancreatitis With Fat Saponification Mimicking Carcinomatosis in an Adolescent: Is It Carcinomatosis or Fat Necrosis?

**DOI:** 10.7759/cureus.105504

**Published:** 2026-03-19

**Authors:** Marcia Mejia, Dellys Adriana Alvarez Alvarado, Ana Maria Trujillo Gómez

**Affiliations:** 1 Radiology, Universidad de Antioquia, Medellin, COL; 2 Pathology, Clínica Las Américas AUNA, Medellin, COL; 3 Radiology, Clínica Las Américas AUNA, Medellin, COL

**Keywords:** acute pancreatitis, children, fat necrosis, fat saponification, peritoneal carcinomatosis

## Abstract

Acute pancreatitis, the most common pancreatic disease in children (1-13 cases per 100,000 children per year), uncommonly presents with extrapancreatic fat necrosis and saponification. This uncommon manifestation can radiologically and intraoperatively resemble peritoneal carcinomatosis or neoplasms, often necessitating histopathological confirmation. Integrating clinical and radiological findings is crucial for accurate diagnosis and to avoid unnecessary interventions. We present a case of an adolescent with acute pancreatitis and extrapancreatic fat saponification mimicking carcinomatosis (with histopathological confirmation), highlighting key imaging features.

## Introduction

Acute pancreatitis is the most common pancreatic disease in children, and its prevalence has increased in recent decades (1-13 cases per 100,000 children per year) likely due to increased clinical awareness, improved diagnostic tools (imaging and testing), and the rise in risk factors such as childhood obesity [1]. It is caused by the activation of pancreatic enzymes that self-digest pancreatic tissue and trigger a systemic inflammatory response. It has multiple etiologies that vary from those seen in adults and include biliary tract disorders, trauma, and medications, among others [2]. For diagnosis, two of three criteria must be met: abdominal pain consistent with the suspicion, elevated serum amylase or lipase >3 times the upper limit of normal, and findings consistent with pancreatitis on diagnostic imaging. The clinical manifestations are mainly epigastric abdominal pain and vomiting, but they can be nonspecific, especially in young children [3].

Pancreatitis is classified as interstitial/edematous or necrotizing, with necrosis occurring in <10% of cases [1]. Necrotizing pancreatitis can rarely manifest with fat saponification of extrapancreatic fat, in some cases acquiring an appearance similar to neoplasms or peritoneal carcinomatosis both on radiological images and intraoperatively [4].

Diagnostic imaging is extremely useful in acute pancreatitis, not only for diagnosis, but also for determining the etiology and possible complications [2]. Extrapancreatic fat saponification is a rare complication presenting a diagnostic challenge and potentially requiring histopathology [4]. Integrating clinical findings and recognizing some key findings in radiological images is essential for correct diagnosis and avoiding unnecessary interventions.

We present a case of an adolescent patient with acute pancreatitis and extrapancreatic fat necrosis mimicking peritoneal carcinomatosis, emphasizing key imaging findings.

## Case presentation

A 14-year-old female patient presented with a three-day history of epigastric pain, persistent vomiting (over 10 episodes), and oral intolerance. On admission, her vitals were stable (BP 110/72 mmHg, 90 bpm, 37.2ºC), but she exhibited mild dehydration and epigastric tenderness without peritoneal signs. Lab results revealed elevated amylase, normal hemoglobin, elevated inflammatory markers and liver function tests (Table 1).

**Table 1:** Laboratory tests with results in the middle column and reference values in the last column. CRP: C-reactive protein; ESR: Erythrocyte sedimentation rate; ALT: Alanine aminotransferase; AST: Aspartate aminotransferase; GGT: Gamma-glutamyl transferase.

Laboratory tests	Results	Reference values
Hemoglobin	15.1	14-18 g/dL
Amylase	1097	20-160 U/l
Leukocytes	14.2	4.4-12 x 10^3^/µL
Neutrophils	12.3	1.5-7.26 x 10^3^/µL
CRP	26	<1 mg/dL
ESR	23	Males <15
ALT	223	4-42 U/L
AST	68	0-35 U/L
GGT	288	8-38 U/L
Alkaline phosphatase	131	30-120 U/L
Total bilirubin	0.87	0.3-1 mg/dL

**Figure 1:**
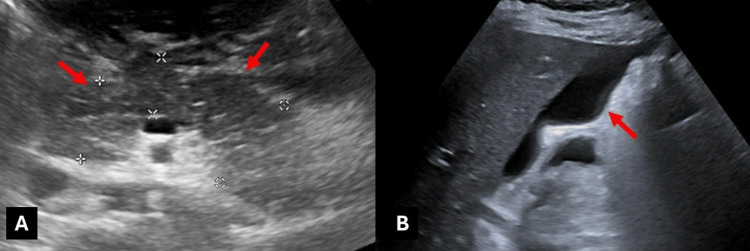
Abdominal ultrasound images showing increased thickness and decreased echogenicity of the pancreatic parenchyma (arrows in A), with free fluid in the abdomen predominantly around the liver (arrow in B).

Abdominal ultrasound showed pancreatic enlargement and altered echogenicity with free abdominal fluid, though visualization was limited by intestinal gas (Figure 1).

**Figure 2:**
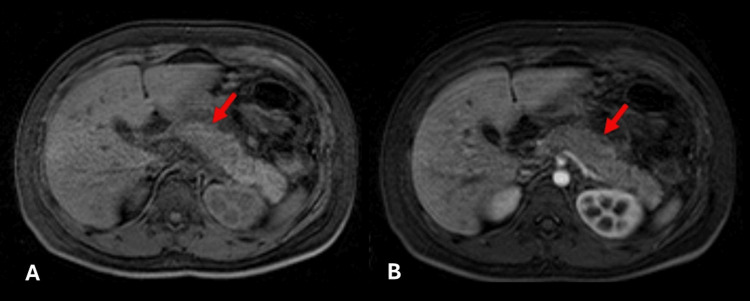
Contrast-enhanced MRI (abdomen): Axial T1-weighted sequence (A) shows increased pancreatic thickness and heterogeneous hypointense signal (red arrow). Arterial phase contrast-enhanced axial T1-weighted sequence (B) shows hypointense pancreatic signal (red arrow).

**Figure 3:**
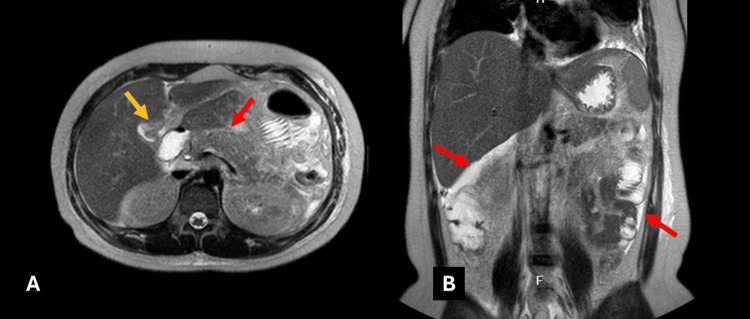
Contrast-enhanced MRI (abdomen): Axial T2-weighted sequence (A) shows mild pancreatic hyperintensity (red arrow) and biliary sludge (yellow arrow). Coronal T2-weighted sequence (B) shows free abdominal fluid (red arrows).

A diagnosis of acute pancreatitis was made, and it was decided to perform a contrast-enhanced MRI to better characterize the bile duct and evaluate possible etiology and complications. Contrast-enhanced MRI revealed diffuse pancreatic enlargement with decreased T1 signal and heterogeneous enhancement, mild T2 hyperintensity, ascites, and biliary sludge (Figures 2, 3).

**Figure 4:**
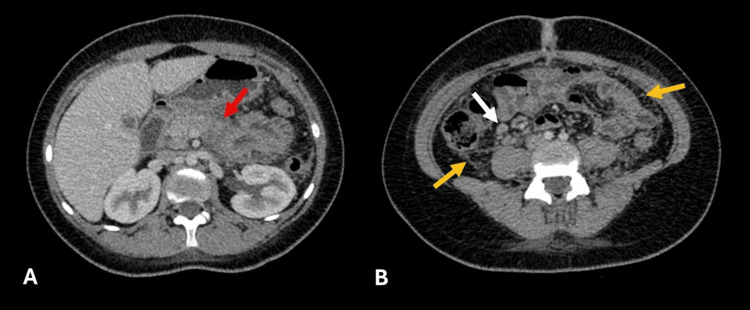
Contrast-enhanced CT (abdomen): Increased pancreatic thickness with heterogeneous enhancement (A, red arrow). Peritoneal and mesenteric micronodules mimicking carcinomatosis (B, yellow arrows), with reactive mesenteric lymph nodes (B, white arrow).

Based on these findings, the pancreatitis was classified as interstitial/edematous, with bile sludge as the possible cause. It was decided to perform a laparoscopic cholecystectomy, and during surgery, widespread whitish peritoneal implants less than 5 mm in size were found, resembling peritoneal carcinomatosis or fat necrosis (intraoperative images not available), prompting biopsy. Post-operative CT showed persistent pancreatitis with abdominopelvic free fluid and extensive peritoneal micronodules mimicking carcinomatosis, along with reactive mesenteric lymph nodes and no obvious primary neoplastic lesions (Figure 4).

**Figure 5:**
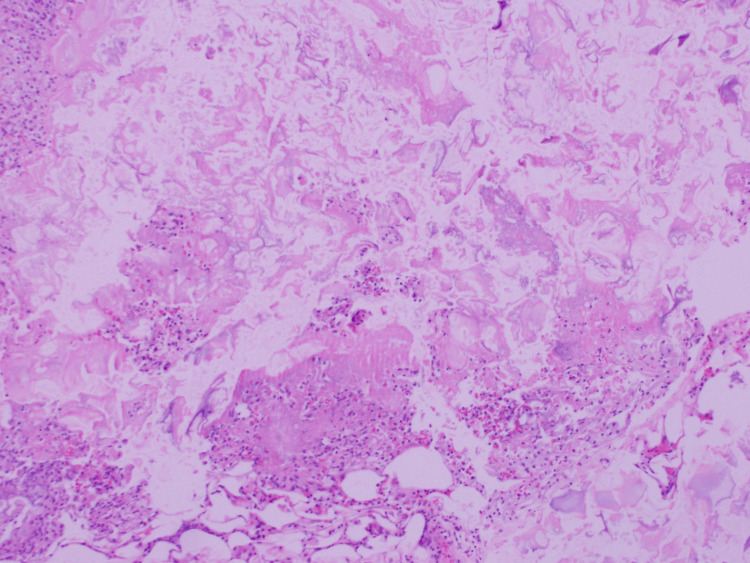
Peritoneal biopsy (H&E): Fibro-adipose tissue with fat necrosis foci surrounded by vacuolated histiocytes and mixed inflammatory infiltrate (plasma cells, lymphocytes, polymorphonuclear neutrophils). No microcalcifications, granulomas, or pathogenic microorganisms identified.

Histopathological results revealed foci of fat necrosis with adjacent inflammatory infiltrates, without evident calcium deposits; no findings suspicious for malignancy were detected (Figure 5).

Based on the above, a final diagnosis of acute pancreatitis with micronodular extrapancreatic fat saponification was made. The patient improved with medical management and was discharged.

## Discussion

Acute pancreatitis is an inflammatory and potentially reversible condition of the pancreas caused by the activation of enzymes within the pancreatic parenchyma. It is more common in adults, but in recent decades, there has been an increase in the pediatric population, with an incidence of 1-13 cases per 100,000 annually [1]. Pancreatitis in children behaves differently than in adults, with different etiologies, clinical presentation, and prognosis. It typically manifests with epigastric abdominal pain and vomiting, although especially in patients under three years of age, symptoms may be nonspecific and include abdominal distension, fever, vomiting, and irritability [1,2]. For diagnosis, at least two criteria defined by INSPPIRE (International Study Group of Pediatric Pancreatitis: In Search for a Cure) must be met: abdominal pain suggestive of or consistent with pancreatitis, serum amylase or lipase at least three times above the upper limit of normal (IU/mL), and imaging findings consistent with pancreatitis [2].

Etiologies include biliary disorders (cholelithiasis, biliary sludge), medications (asparaginase, valproic acid, trimethoprim-sulfamethoxazole, mercaptopurine, mesalamine, and corticosteroids, among others), trauma (accidental/non-accidental), autoimmunity, and idiopathic causes. There are also other risk factors such as anatomical abnormalities (pancreas divisum, pancreaticobiliary maljunction, annular pancreas, choledochal cyst, aberrant bile ducts), sepsis, inflammatory bowel disease, lupus, metabolic disorders, and viral infections, among others [1-3].

The Atlanta classification was created to facilitate the classification and management of adult patients with acute pancreatitis, but it has been extrapolated to the pediatric population. It divides pancreatitis into two groups (interstitial/edematous and necrotizing), with different degrees of severity (defined according to the presence of organ failure and/or complications) and different types of fluid collections (according to the time of evolution and the presence or absence of necrosis) [1].

Interstitial or edematous pancreatitis is the most common subtype (90-95% of cases), with the remainder corresponding to necrotizing pancreatitis [2]. Necrotizing pancreatitis can affect the pancreatic parenchyma, extrapancreatic fat, or both, with mixed involvement being more common [4].

The pathophysiological mechanism of extrapancreatic necrosis is caused by the release of enzymes that self-digest the pancreas and extrapancreatic fat, with an increase in the inflammatory response, migration of macrophages to fatty tissue, and rupture of adipocyte cell membranes. This leads to the release and hydrolysis of triglycerides, generating free fatty acids. These fatty acids combine with serum calcium and precipitate in a saponification reaction [4-6].

Saponification usually occurs in peripancreatic fat, retroperitoneum, omentum, mesentery, and transverse mesocolon, but unusual locations include peritoneal fat (simulating carcinomatosis) and extra-abdominal fat (subcutaneous tissue, periarticular tissues, mediastinum) [5-7].

Radiological imaging is crucial for diagnosis, etiology determination, and complication detection. Ultrasound is the initial study for suspected pediatric pancreatitis, avoiding radiation and identifying biliary abnormalities. CT and MRI are reserved for detecting complications or when ultrasound is inadequate, ideally performed >48 hours after symptom onset for improved necrosis detection [1-3].

Ultrasound findings of acute pancreatitis will be observed in 30-52% of patients and include an increase in the size of the pancreas (focal or diffuse), a decrease or increase in parenchymal echogenicity, poor contour definition, dilation of the main duct, fluid, and peripancreatic collections. Contrast-enhanced CT will show findings in up to 47-75% of patients, including: increased pancreatic size (focal or diffuse), parenchymal hypodensity or heterogeneous enhancement, poorly defined contours, peripancreatic fat stranding, peripancreatic fluid, and thickening of the retroperitoneal fascia; however, tomography has limited usefulness for assessing the biliary tree. Magnetic resonance imaging will have better contrast resolution than other imaging methods, making it more sensitive for detecting edema and hemorrhage. It will also allow for better assessment of the biliary tree, detecting anatomical abnormalities and stones. MRI findings include enlargement of the pancreas, heterogeneous hypointense parenchyma on T1-weighted images and hyperintense on T2-weighted images, hypoenhancement, diffusion restriction, and peripancreatic fluid (best visualized on T2-weighted images) [1-3]. Pancreatic necrosis will manifest as an absence of parenchymal enhancement in both CT and MRI [7, 8].

Extrapancreatic necrosis can manifest as multiple nodules (simulating carcinomatosis) or masses (simulating neoplasms), sometimes requiring histological analysis. These manifestations usually occur after the acute phase of pancreatitis has resolved, but in some patients, they may occur earlier. Differentiating fat saponification from carcinomatosis radiologically can be difficult; however, in saponification, lesions show less, more heterogeneous, and delayed enhancement, with occasional calcifications [6-9].

Pediatric acute pancreatitis treatment is supportive: fluids, early nutrition (within 48 hours), pain control, etiology identification, reversible cause management, and complication management. Antibiotics are reserved for necrosis and infection. Extrapancreatic necrosis management is typically conservative unless infection occurs [1-3].

Our patient's acute pancreatitis with extrapancreatic necrosis and fat saponification presented with radiological and intraoperative findings mimicking peritoneal carcinomatosis. However, their appearance during the acute phase (unlike typical reports) caused diagnostic uncertainty, necessitating histopathological confirmation. Conservative management led to a positive clinical outcome.

## Conclusions

Extrapancreatic fat saponification, a rare complication of acute pancreatitis, can mimic carcinomatosis on imaging. Accurate diagnosis relies on recognizing key radiological features combined with clinical correlation to avoid unnecessary procedures. We present a case of an adolescent with acute necrotizing pancreatitis and extrapancreatic fat saponification. Imaging mimicked peritoneal carcinomatosis but occurred early in the disease course and required histopathological confirmation.
